# AquaMamba: Selective state space modeling for underwater scene segmentation in marine robotics

**DOI:** 10.1371/journal.pone.0360659

**Published:** 2026-11-11

**Authors:** Shuxi Chen, Jianlin Qiu, Yiyang Sun, Haifei Zhang, Hao Chen

**Affiliations:** 1 School of Yonyou Digital and Intelligence, Nantong Institute of Technology, Nantong, China; 2 Division of Information and Communication Convergence Engineering, Mokwon University, Daejeon, Korea; 3 School of Information Science and Technology, Nantong University, Nantong, China; 4 School of Information Technology, Nantong Institute of Technology, Nantong, China; 5 School of Electrical Engineering and Computer Science, Royal Institute of Technology (KTH), Stockholm, Sweden; Institut de Robotica i Informatica Industrial, SPAIN

## Abstract

Underwater scene segmentation is challenging because image degradation and domain shift vary greatly in marine environments, and the behavior of selective state-space models (SSMs) in this environment remains insufficiently characterized. We propose AquaMamba, a ResNet34-UNet segmentation framework that replaces the bottleneck operator with stacked Mamba layers, and compare it with five established CNN-based methods and a constructed Transformer-bottleneck baseline in the SUIM benchmark. AquaMamba achieves an mIoU of 0.669, 1.9 percentage points higher than the controlled Trans-UNet baseline (0.650), while using fewer parameters (29.5M vs. 33.9M). DeepLabV3 + remains the strongest overall model, reaching 0.712 mIoU. On the unlabeled UIEB dataset, AquaMamba shows the highest confidence retention among four representative models, retaining 93.5% of its source-domain prediction confidence. Due to the lack of semantic annotation in UIEB, this result reflects prediction confidence rather than target-domain segmentation accuracy. Scale- and boundary-specific diagnostics within the selected representative subset further show complementary behavior among AquaMamba, Trans-UNet and DeepLabV3 + : AquaMamba is competitive in several large object categories and obtains the lowest boundary error point estimate, while DeepLabV3 + remains stronger for human divers and plant/seagrass, and AquaMamba is limited on the reported small-RO subset. These findings position AquaMamba as a controlled SSM baseline for underwater semantic segmentation and emphasize the need for future SSM designs to better integrate multi-scale and high-resolution features.

## 1. Introduction

Underwater visual perception is critical for autonomous hull inspection, marine ecology monitoring, and underwater robotics [1]. However, underwater images are inherently degraded by wavelength-dependent light absorption, particle scattering, and color distortion [2,3], making semantic segmentation in this domain uniquely challenging.

Convolutional Neural Networks (CNNs) have been the dominant paradigm for underwater segmentation [3,4], yet two fundamental limitations motivate the exploration of alternative architectures. First, CNNs rely on local convolutions with bounded receptive fields [5], which hampers their ability to interpret degraded scenes where local cues are obscured by turbidity or color cast. Second, Vision Transformers [6,7] can capture global dependencies but full-resolution attention has quadratic complexity O(L^2^) in the token-sequence length, which can become costly for high-resolution underwater robotic perception and inspection imagery.

Selective SSMs, exemplified by Mamba [8], offer an alternative by combining linear-time complexity with global receptive fields. Notably, Mamba’s selective mechanism enables input-dependent filtering, a property that is potentially useful for modeling semantic content under spatially varying degradation in underwater images. Although recent studies have applied Mamba to medical imaging [9] and visual representation learning [10], and Mamba-based models have recently been introduced for underwater image enhancement and underwater instance segmentation [11–13], their application in underwater semantic segmentation remains largely unexplored to our knowledge. This field faces unique challenges, including severe image degradation, extreme class imbalance, and domain shift across varying water conditions [14]. In section 2.2, we review these emerging SSM-based underwater methods and clarify how our contributions differ from them.

Existing underwater segmentation approaches fall into three categories: (1) auxiliary-input methods that require depth sensors or multi-modal data [3], limiting deployment flexibility; (2) attention-based methods that achieve strong performance but at quadratic computational cost and high parameter counts [7,15]; and (3) standard CNN methods [4] that provide less explicit long-range sequence modeling. Our proposed AquaMamba is designed to mitigate these constraints: it operates on RGB-only input without auxiliary sensors, uses a linear-complexity sequence operator at the bottleneck, and provides global-context modeling through selective state space modeling.

In addition, as far as we know, the cross-domain confidence behavior of underwater segmentation models has not been systematically evaluated. This is an important consideration for Autonomous Underwater Vehicle (AUV) perception systems that traverse diverse marine environments. Cross-domain analysis shows that AquaMamba maintains a higher predictive confidence on unlabeled target domains, which may be related to its content adaptive filtering mechanism.

This paper addresses three research questions: (1) How does Mamba compare to Transformers and CNNs for underwater segmentation? (2) What are Mamba’s specific strengths and limitations in this domain? (3) How does prediction confidence change under an unlabeled domain shift?

Our main contributions are summarized as follows:

1. We propose AquaMamba, which, to our knowledge, is among the first architectures to apply selective state space modeling to the segmentation of underwater semantic scenes. Although its pretrained ResNet encoder, Mamba bottleneck and UNet decoder draw on existing designs, our main contribution is application-focused: we adapt and evaluate SSM-based global-context modeling for degraded, class-imbalanced underwater scenes.
2. Through a controlled comparison with five established CNN-based methods and a constructed Transformer-bottleneck baseline on the SUIM benchmark [4], we evaluate selective SSMs for underwater semantic segmentation. AquaMamba is 1.9 percentage points higher than the constructed Transformer-bottleneck baseline in mIoU, while the full benchmark also shows that multi-scale modeling remains an important direction for further improvement.
3. We evaluate cross-domain prediction confidence on the unlabeled UIEB dataset and explicitly distinguish confidence retention from target-domain segmentation accuracy. The analysis describes how model confidence changes under domain shift and acknowledges that high-confidence predictions may still be incorrect.

## 2. Related work

### 2.1. Underwater image segmentation

A substantial part of underwater vision research has focused on image enhancement as preprocessing [16,17]. Islam et al. [4] introduced the SUIM dataset with 1,635 annotated images (1,525 training / 110 test) across 8 semantic categories, establishing benchmarks using ResNet and VGG backbones. Subsequent methods employ UNet variants [18], attention-gated networks [19] and multi-scale fusion [20], but most still rely primarily on convolutional feature extraction and provide less explicit long-range sequence modeling. In addition to SUIM, other underwater segmentation datasets have recently emerged, such as the UIIS dataset for underwater instance segmentation [21]. Our work complements this line of research by evaluating selective state-space modeling and a controlled Transformer bottleneck for underwater semantic segmentation.

### 2.2. SSMs for vision

SSMs originated from control theory and have recently shown strong promise for sequence modeling. Gu et al. [22] introduced linear state-space layers combining recurrent, convolutional, and continuous-time models. Mamba [8] enhanced SSMs with selective mechanisms, enabling input-dependent parameter computation and hardware-aware sequence modeling, and achieved competitive performance with Transformers [23] in language modeling. For vision tasks, Vision Mamba [10] applies bidirectional state space modeling for efficient visual representation learning. SegMamba [9] extends it to 3D medical image segmentation. Recently, SSM/Mamba architectures have also begun to appear in the underwater domain, mainly for underlying tasks such as underwater image enhancement and recovery and underwater instance segmentation [11–13]. These works prove the potential of selective state space modeling in underwater vision, but they mainly target image quality recovery or instance-level masks rather than dense semantic scene segmentation, and they do not pay attention to the cross-domain confidence retention for semantic segmentation. In contrast, our research focuses on pixel-level semantic segmentation of underwater scenes, compares the SSM bottleneck with a controlled Transformer bottleneck under the same ResNet34-UNet setting, using the same ResNet34 backbone network to evaluate the CNN baseline, and providing tailor-made diagnosis and cross-domain confidence analysis for marine robot deployment. Aside from these emerging efforts, most vision SSM studies have been developed and evaluated outside underwater semantic segmentation, whereas underwater vision presents distinct challenges, including severe degradation, extreme class imbalance, and domain shift across water types. How SSM-based models behave under these conditions therefore remains insufficiently characterized.

### 2.3. Multi-scale segmentation architectures

DeepLabV3+ [24] uses Atrous Spatial Pyramid Pooling (ASPP) to capture multi-scale context through parallel atrous convolutions at different dilation rates. FPN [20] employs top-down pathways with lateral connections. UNet++ [25] introduces dense skip connections for multi-scale feature aggregation. Pure Transformer-based segmentation architectures, such as Swin-Unet [15], have also achieved strong results in medical imaging although their role in underwater semantic segmentation remains less established. These architectures prompt us to further analyse whether multi-scale feature aggregation is still important compared with bottleneck-level sequence modeling.

## 3. Method

### 3.1. Architecture overview

Fig 1 illustrates the AquaMamba architecture, which combines CNN-based local feature extraction with Mamba-based global-context modeling.

**Fig 1:**
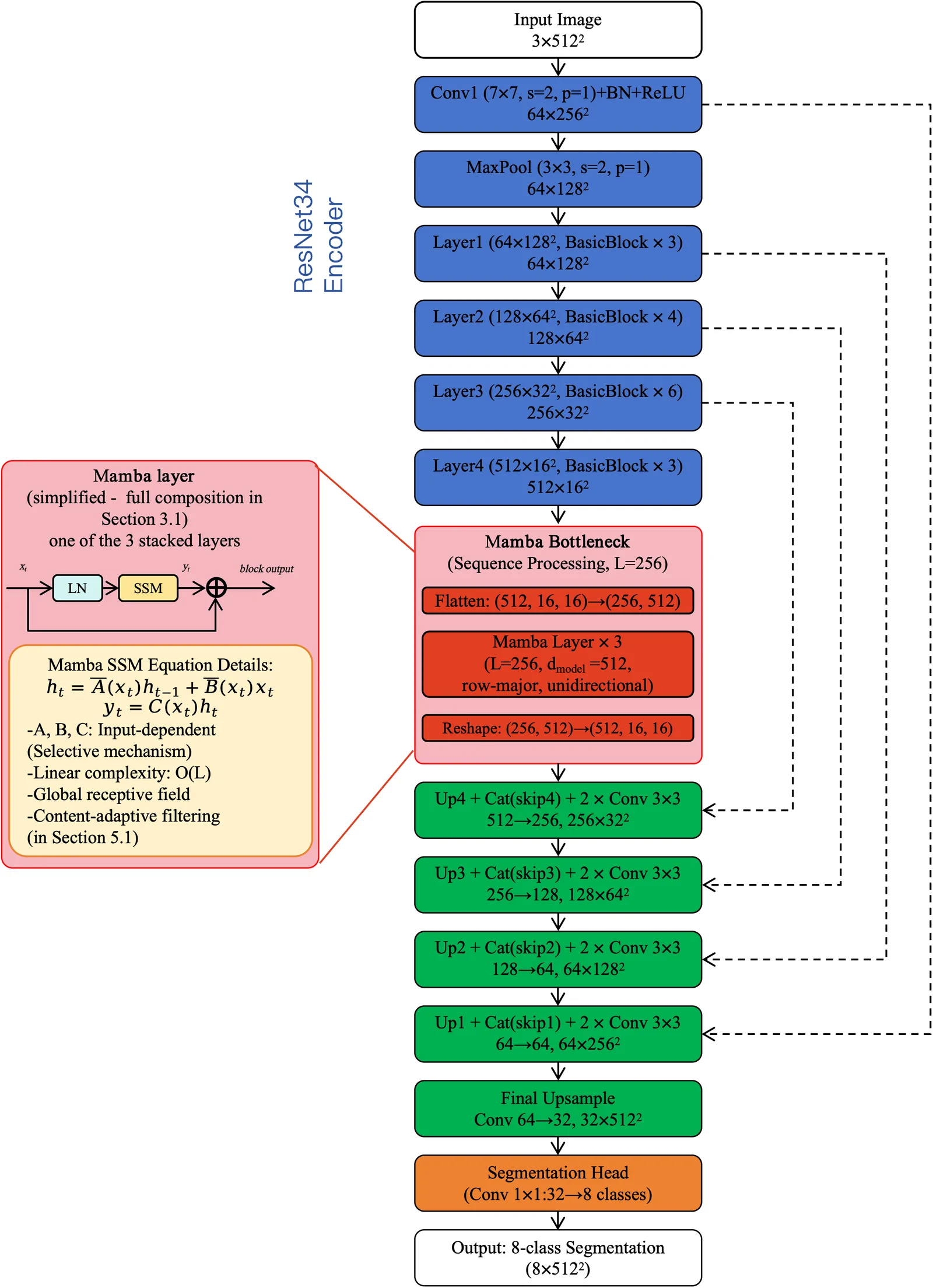
Overview of the AquaMamba architecture. Shapes are shown as channels × resolution^2^. The ResNet34 encoder (blue) downsamples the input to a 512 × 16^2^ bottleneck feature map, which is flattened by the Mamba bottleneck (red) into L = 256 row-major tokens before being reshaped for decoding. The UNet decoder (green) restores the prediction to the input resolution using encoder skip connections. The inset shows a simplified Mamba layer and full implementation details are provided in Section 3.1.

**Encoder**. We employ ResNet34 [26] pretrained on ImageNet [27] to extract hierarchical features through a stem (7 × 7 conv, stride 2, producing 64 channels at H/2) followed by 4 residual stages at {H/4, H/8, H/16, H/32} with channels {64, 128, 256, 512}. We use ResNet34 instead of a larger encoder to maintain a moderate parameter budget on a limited SUIM training set and to maintain a fair comparison between the evaluated architectures.

**Mamba Bottleneck.** At the deepest encoder level (512 channels, H/32 × W/32 resolution, i.e., 16 × 16 for a 512 × 512 input), we apply 3 stacked Mamba layers. This bottleneck placement is intended to provide global-context modeling with limited computational cost: operating on the smallest feature map yields a sequence length of only 256 tokens, 1024 × fewer than full-resolution 512 × 512 tokenization.

Each Mamba layer implements selective state space dynamics [8], as shown in Eq (1):


$$\begin{array}{c} {\text{h}}_{t}=\overset{―}{A}\left(x_{t}\right){\text{h}}_{t-\text{1}}+\overset{―}{B}\left(x_{t}\right)x_{t},y_{t}=C\left(x_{t}\right){\text{h}}_{t} \end{array}$$ (1)

where $\overset{―}{A}$, $\overset{―}{B}$ and C are input-dependent parameters, enabling content-aware filtering. This selectivity is potentially useful for underwater scenes because it allows the model to adapt its filtering behavior to spatially varying content and degradation patterns, such as turbidity and color distortion [17]. Operating at the *H*/32 resolution effectively compresses the spatial sequence length, which is well suited to SSM’s global-context modeling. However, this stzrong down-sampling also creates an information bottleneck that can reduce fine-grained high-frequency details.

In Eq (1), $\text{x}\in {\text{R}}^{{\text{d}}_{\text{model}}}$ denotes the token at position *t* of the flattened bo*t*tleneck sequence (*t* = 1, …, L with L = 256). $\text{h}\in {\text{R}}^{{\text{d}}_{\text{model}}\;\times \;{\text{d}}_{\text{state}}}$ is the hidden sta*t*e and $\text{y}\in {\text{R}}^{{\text{d}}_{\text{model}}}$ the output. $\overset{―}{\text{A}}\left(\text{x}\right)$ and $\overset{―}{\text{B}}(\text{x})\in {\text{R}}^{{\text{d}}_{\text{model}}\;\times \;{\text{d}}_{\text{state}}}$ are the discretized state-transition and input matrices, obtained from the continuous-time parameters through a zero-order hold with an input-dependent step size Δ(x), and $\text{C}\left(\text{x}\right)\in {\text{R}}^{{\text{d}}_{\text{model}}\;\times \;{\text{d}}_{\text{state}}}$ is the output matrix. The dependence of Δ, B and C on *x* is what makes the model selective: the effective filter is recomputed at every spatial position instead of being fixed after training. A signal-processing interpretation of this mechanism is provided in Section 5.1.

**Implementation of the bottleneck.** The bottleneck feature map $\text{x }\in {\text{ R}}^{\text{512}\times \text{16}\times \text{16}}$ is flattened in row-major order into a sequence of L = 256 tokens with ${\text{d}}_{\text{model}}\;=\text{ 512}$. The sequence is processed by three Mamba blocks and reshaped back to 512 × 16 × 16 before entering the decoder. Each block uses pre-normalization, an input projection with expansion factor E = 2, a depth-wise 1-D convolution with kernel size 4, a selective SSM with state dimension ${\text{d}}_{\text{state}}\;=\text{ 16}$, SiLU gating, an output projection, and a residual connection. The scanning is carried out in one direction in the order of the raster without bidirectional or cross-scan variants.

**Parameter accounting.** The ResNet34 encoder contributes 21.28M parameters, and the UNet decoder contributes 3.15M parameters, giving a shared 24.43M backbone. In depth sequence statistics, about 1.7 million parameters are added to each Mamba layer. The total parameter volume of the measured 1-, 3- and 6-layer configurations is 26.13M, 29.52M and 34.61M respectively. The deployed AquaMamba adopts a 3-layer bottleneck structure with a total parameter volume of 29.52M.

**Decoder.** A symmetric UNet decoder progressively upsamples the bottleneck output back to the input resolution. Each of the four decoder stages applies a 2 × transposed convolution, concatenates the corresponding encoder skip connection [18], and applies two 3 × 3 convolutions, with channel widths 512 → 256, 256 → 128, 128 → 64 and 64 → 64 at resolutions 32^2^, 64^2^, 128^2^ and 256^2^, respectively. The skip connections are taken from Layer3 (256 × 32^2^), Layer2 (128 × 64^2^), Layer1 (64 × 128^2^) and the stem convolution (64 × 256^2^); Layer4 carries no skip connection, since its output is the input of the bottleneck. A final transposed convolution restores the input resolution (64 → 32 channels at 512 × 512), after which a 1 × 1 convolution maps the 32 channels to the 8 semantic categories [28].

### 3.2. Training strategy

**Loss Function**. We combine weighted Cross-Entropy and Dice losses [29], as defined in Eq (2).


$$\begin{array}{c} \text{L}={\text{L}}_{WCE}\left(p,\;y\right)+\lambda \cdot {\text{L}}_{Dice}\left(p,\;y\right) \end{array}$$ (2)

where λ = 0.5 balances the two terms.

Class weights are computed as inverse class frequencies to mitigate severe class imbalance; for example, the HD category appears in only approximately 12% of the training images.

**Optimization**. We optimize the model using AdamW [30] with a learning rate of $\text{3}\times {\text{10}}^{-\text{4}}$, cosine annealing with a 5-epoch warmup [31], weight decay of 0.05, and gradient clipping with a maximum norm of 1.0 for training stability.

**Augmentation.** All augmentations are applied instantly and sequentially. First, the random horizontal and vertical flips are applied independently, and the probability of each flip is p = 0.5. Then apply colour jitter (p = 1.0) to each training sample to disturb the brightness, contrast and saturation up to ±30%. After using ImageNet statistics to normalise each channel, apply σ = 0.02 added Gaussian noise to each normalised training tensor (p = 1.0) [32].

## 4. Experiments

### 4.1. Experimental setup

**Dataset** SUIM [4]: 1,525 training and 110 test images across 8 semantic categories, including: Background (BG), Human Divers (HD), Plants/Seagrass (PF), Wrecks/Ruins (WR), Robots/Remotely Operated Vehicles (ROVs) (RO), Reefs/Invertebrates (RI), Fish/Vertebrates (FV), Sea-floor/Rocks (SR). Images are captured in diverse underwater environments with varying turbidity and lighting [33]. We adopt SUIM as our primary benchmark because it is a widely used dataset that provides intensive pixel-level semantic annotations these 8 categories of underwater scenes, so that direct comparisons based on intersection over union (IoU) can be made. There are other underwater datasets, but they are aimed at different tasks or label formats. For example, UIIS [21] provides an instance-level mask for underwater instance segmentation, not a dense semantic label. Therefore, we use SUIM to conduct major quantitative research and leave the broader multi-dataset semantic evaluation for future work.

**Benchmark models**. The full SUIM benchmark includes AquaMamba and six baseline methods spanning three broad architectural paradigms:

*• Standard UNet-based CNNs*: UNet [18], UNet++ [25]

*• Multi-scale CNN*: DeepLabV3+ [24], FPN [20], MA-Net [34]

*• Sequence modeling*: Trans-UNet is a UNet architecture, in which the bottleneck is replaced by a 3-layer Transformer encoder [23] with 8-head multi-head self-attention, a model dimension of 512 and a feed-forward dimension of 2,048. Like AquaMamba, it uses the same 256-token bottleneck sequence and fixed sinusoidal positional encoding, while AquaMamba uses the raster-scanned sequence without explicit positional encoding. This baseline compares Transformer- and SSM-based bottleneck sequence modeling under the same ResNet34-UNet setting. Each Transformer layer contains about 3.15 million parameters, approximately 1.9 times the number of parameters in the Mamba block it replaces.

All other baseline models also use pretrained ResNet34 encoders on ImageNet [26,27] to ensure that their performance differences are mainly reflected in the decoder design, not in the selection of the backbone network.

Representative-model selection for extended analyses. Table 1 reports the full seven-architecture benchmark. The cross-domain confidence analysis includes four representative models (UNet, DeepLabV3 + , Trans-UNet, and AquaMamba), with results reported in Table 4 and visualized in Fig 2(a) and (b). The object-size and boundary diagnostics (Table 5 and Fig 3) and the class-wise IoU difference analysis (Fig 4) focus on DeepLabV3 + , Trans-UNet, and AquaMamba. For the object-size, boundary-error, and class-wise IoU difference analyses, these three models were selected to compare the strongest full-benchmark baseline, the controlled Transformer counterpart, and the proposed SSM model. UNet, DeepLabV3 + , Trans-UNet, and AquaMamba represent standard CNN, multi-scale CNN, Transformer-bottleneck, and SSM-bottleneck settings, respectively. UNet is used as the standard CNN representative because it is the original encoder-decoder reference architecture, whereas UNet++ is retained in the full benchmark but not used as the representative model because its nested skip connections add a stronger refinement mechanism. Therefore, conclusions from these extended analyses are interpreted as observations within the selected representative subset, not as claims covering all seven architectures.

**Table 1:** Comparison on the SUIM test set. All methods use ResNet34 backbones initialized with ImageNet-pretrained weights. Best values are shown in bold and second-best values are underlined. Class abbreviations: BG = Background, HD = Human Divers, PF = Plants/Seagrass, WR = Wrecks/Ruins, RO = Robots/ROVs, RI = Reefs/Invertebrates, FV = Fish/Vertebrates and SR = Sea-floor/Rocks.

Type	Method	Params	BG	HD	PF	WR	RO	RI	FV	SR	mIoU	FPS
Multi-scale	DeepLabV3+	22.4M	**.876**	.720	**.407**	.776	.780	**.694**	**.748**	**.693**	**.712**	**283**
	FPN	23.2M	.874	**.761**	.280	.775	.791	.685	.711	.674	.694	178
UNet-based	UNet++	26.1M	.854	.754	.260	**.809**	**.799**	**.694**	.742	.669	.698	189
	UNet	24.4M	.859	.667	.331	.780	.772	.671	.699	.637	.677	265
	MA-Net	31.8M	.870	.465	.240	.704	.592	.578	.601	.647	.587	187
Sequence	**AquaMamba (Ours)**	29.5M	.856	.667	.338	.744	.735	.667	.711	.637	.669	203
	Trans-UNet ^†^	33.9M	.867	.631	.257	.730	.766	.653	.632	.661	.650	225

† Trans-UNet is the constructed Transformer-bottleneck baseline described in Section 4.1.

**Fig 2:**
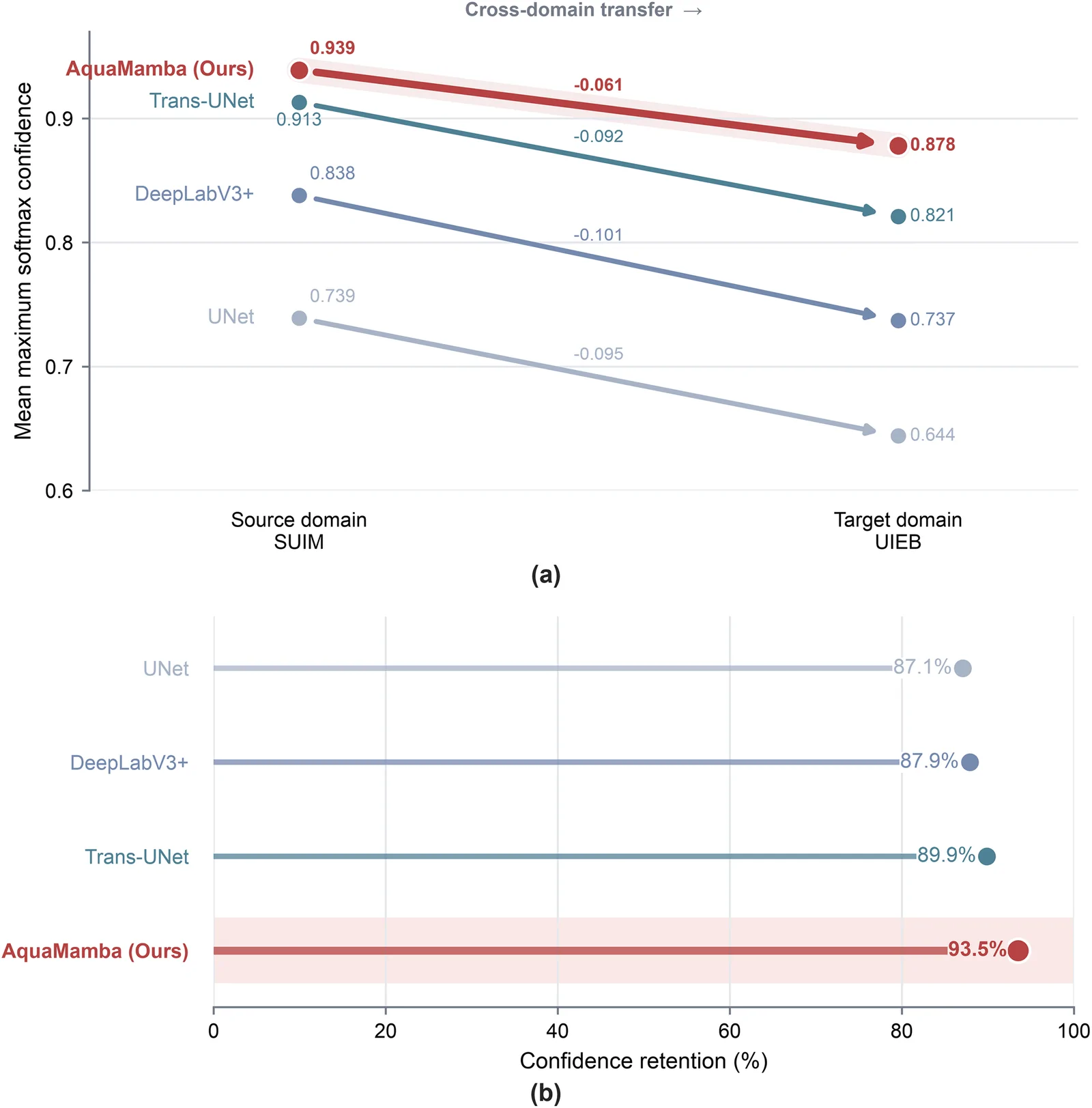
Cross-domain prediction confidence and retention across four representative models. (a) Mean maximum softmax confidence of UNet, DeepLabV3 + , Trans-UNet, and AquaMamba on SUIM (source domain) and UIEB (unlabeled target domain). The slope annotations indicate confidence changes (UIEB minus SUIM), with more negative values representing larger declines. (b) Confidence retention, calculated as the ratio of mean confidence on UIEB to that on SUIM and expressed as a percentage. Higher retention indicates greater preservation of prediction confidence under domain shift but does not establish higher target-domain segmentation accuracy.

**Fig 3:**
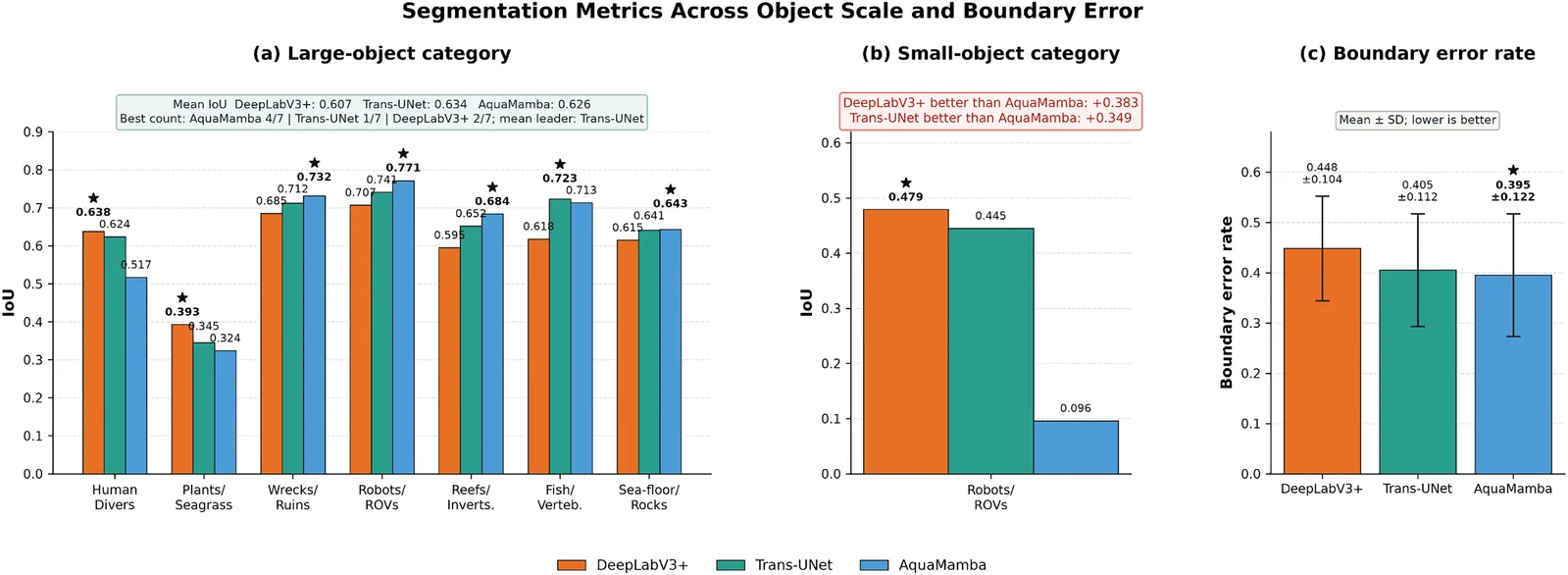
Diagnostic analysis comparing AquaMamba, Trans-UNet and DeepLabV3+ on the SUIM test set. Orange, green and blue bars denote DeepLabV3 + , Trans-UNet and AquaMamba, respectively. (a) Large object IoU by class: AquaMamba is highest on 4 of 7 categories (WR, RO, RI and SR), DeepLabV3 + is highest on HD and PF, and Trans-UNet is highest on FV. (b) Small object IoU for RO, the only small object category retained for category-level reporting in this analysis; other small-object categories are omitted because the available small connected components were too sparse for a stable estimate. (c) Boundary-error rate, where lower is better; error bars denote per-image standard deviation over the 110 test images.

**Fig 4:**
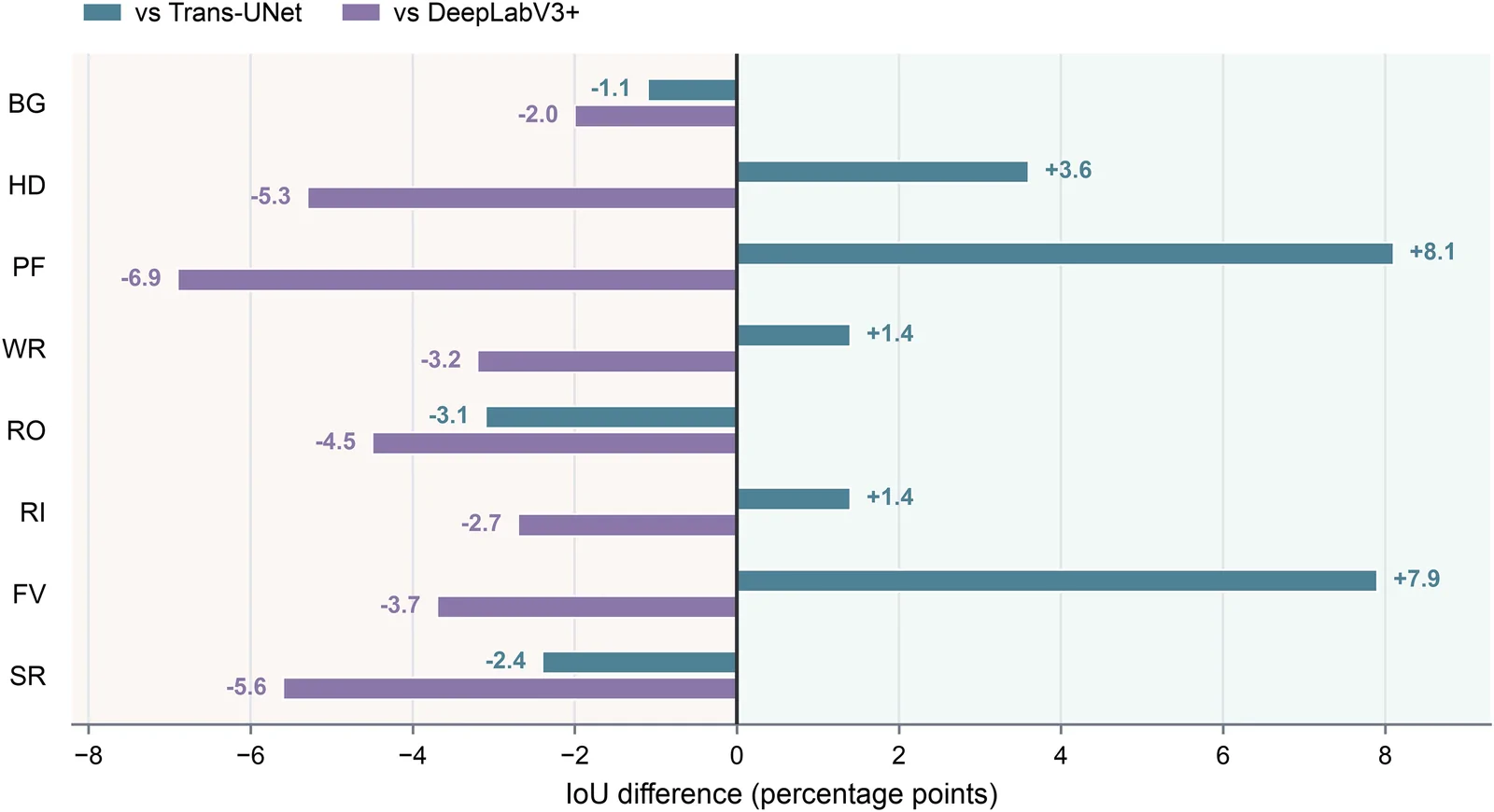
Class-wise IoU differences between AquaMamba and two representative baselines on SUIM. Differences are calculated as IoU(AquaMamba) minus IoU(baseline) for each of the eight semantic categories on the SUIM test set and are expressed in percentage points. Positive values indicate higher IoU for AquaMamba, whereas negative values indicate higher IoU for the corresponding baseline. The two baselines are Trans-UNet and DeepLabV3 + , and category abbreviations follow Table 1.

**Evaluation metrics.** We evaluate the models using mean IoU (mIoU), class-wise IoU and inference speed in frames per second (FPS) [28,35]. All models are evaluated using the same input resolution and hardware configuration. Because the evaluated models use the same ResNet34 backbone and input size, FPS differences primarily reflect variations in decoder and bottleneck design, implementation details and operator efficiency. FPS is reported as a practical measure of computational throughput.

**Experimental environment.**. The experiments are implemented on a server equipped with a single NVIDIA RTX 4090 GPU and 16 Intel Xeon Gold 6430 vCPUs, running Ubuntu 22.04, Python 3.12, PyTorch 2.3.0, and CUDA 12.1. For FPS measurement, the input resolution is fixed at 512 × 512 and the batch size is set to 4. After 50 warm-up iterations, inference speed is measured over the SUIM test set under the same configuration for all models. The reported FPS values characterize throughput in the evaluated server-GPU environment and should not be interpreted as validated onboard AUV performance.

### 4.2. In-Domain results

Table 1 presents the comparison results.

**Finding 1**: AquaMamba outperforms the constructed Transformer bottleneck under the evaluated setting. AquaMamba achieves an mIoU of 0.669, exceeding the constructed Trans-UNet baseline by 1.9 percentage points (0.650), while using 13% fewer parameters (29.5M vs. 33.9M). These results indicate that, within the evaluated ResNet34-UNet framework, the Mamba bottleneck provides a more favorable parameter-accuracy trade-off than the Transformer bottleneck. However, a lower parameter count does not necessarily imply reduced inference latency or memory consumption, and this comparison is limited to the specific implementations evaluated here. Both sequence-modeling variants also outperform MA-Net on this benchmark, although these results do not isolate global-context modeling as the sole cause of the improvement.

**Finding 2**: effective multi-scale feature extraction remains important. DeepLabV3 + achieves the best overall performance (0.712 mIoU), consistent with the benefit of ASPP’s multi-scale atrous convolutions for capturing context at dilation rates of 6, 12 and 18. The 0.043 gap between DeepLabV3+ and AquaMamba is most visible in deformable categories and the reported small-RO subset, where multi-scale receptive fields and high-resolution feature preservation appear particularly important. This observation suggests a possible direction for improvement: future SSM architectures may benefit from integrating multi-scale mechanisms with global modeling.

**Finding 3:** model complexity should be interpreted relative to data scale. The two largest models, MA-Net (31.8M, 0.587) and Trans-UNet (33.9M, 0.650), rank among lower-performing models, while the compact DeepLabV3 + model (22.4M) ranks first. This suggests that, with limited underwater data, higher-capacity models do not necessarily generalize better and that architectural design matters more than raw parameter count. This observation is consistent with previous reports that high-capacity models can overfit small segmentation datasets [36]. However, overfitting is not established as the only explanation in our experiments; optimization difficulty and implementation details may also contribute.

### 4.3. Per-class analysis

Table 1 also reveals distinct class-specific performance patterns. AquaMamba remains competitive in the visually challenging and example-limited HD (0.667 IoU) and PF (0.338 IoU) categories, although Table 1 alone does not identify the mechanism behind this pattern. When evaluated across all scales, DeepLabV3 + achieves high accuracy in structurally prominent categories, including RO (0.780 IoU), WR (0.776 IoU) and RI (0.694 IoU), which is consistent with the value of multi-scale receptive fields in in-domain segmentation. As an auxiliary descriptive check, we computed the mean number of predicted categories per image, defined as the number of different semantic categories assigned by the model in an image and then averaged over the test set. UNet predicts an average of 6.4 distinct semantic categories per image, compared with 4.4 for AquaMamba and DeepLabV3 + . Since this auxiliary statistic was not extended to all baselines, we use it only as a descriptive observation and do not draw comparative conclusions from it. This metric describes prediction diversity only; because it is not normalized by the number of true categories in each image, it should not be interpreted as evidence of over-prediction, false-positive rate or segmentation correctness.

### 4.4. Ablation study

We conduct two sets of ablation experiments to examine architectural tendencies under a simplified and controlled optimization protocol. These ablations are intended to compare configurations within each table, rather than to provide directly comparable numbers with the final 50-epoch training protocol in Table 1. All ablation experiments use simplified training (30 epochs, fixed learning rate) for fair comparison. The deployed model uses the full training protocol (50 epochs, warm-up, cosine annealing) achieving 0.669 mIoU which is shown in Table 1.

**Mamba Bottleneck Depth**. Table 2 evaluates the impact of the number of Mamba layer on the bottleneck position. The depth of the evaluation shows an inverted-U pattern. Compared with the no-Mamba baseline (0.497 mIoU), the 1-layer configuration has a smallest gain (0.500 mIoU, + 0.003), the 3-layer configuration has the best performance in the depth of evaluation (0.517 mIoU, + 0.020), while the performance of the 6-layer configuration is lower than the baseline (0.488 mIoU, −0.009). It is worth noting that the 6-layer configuration has the most parameters (34.61M), but the performance is not the best, which shows that performance improvement is not only determined by the number of parameters. We believe that part of the reason for the decline in the performance of the 6-layer configuration is the difficulty of optimization and the limited training set that may lead to overfitting, but we do not claim that overfitting is the only reason. Therefore, we have adopted the 3-layer bottleneck in AquaMamba.

**Table 2:** Ablation on Mamba bottleneck depth under the simplified protocol (30 epochs, fixed learning rate). Among the evaluated depths {0, 1, 3, 6}, D3 gives the highest mIoU and is adopted in AquaMamba.

Configuration	Layers	Params	mIoU
D0: ResNet34-UNet (no Mamba)	0	29.16M	0.497
D1: + Mamba bottleneck, 1 layer	1	26.13M	0.500
D3: + Mamba bottleneck, 3 layers (adopted in AquaMamba)	**3**	**29.52M**	**0.517**
D6: + Mamba bottleneck, 6 layers	6	34.61M	0.488

**Note**: Ablation values are comparable within this table, but it cannot be directly compared with Table 1, because the final model reported in Table 1 adopts the complete training scheme described in Section 3.2. D3 in Table 2 and P2 in Table 3 are the same architecture trained in two independent ablation runs, and the difference between the two measurements is 0.005 mIoU. The number of parameters of D1 is less than D0, because the Mamba bottleneck convolutional layer replaces the two 3 × 3 bottleneck convolution layers in the ResNet34-UNet baseline model instead of adding them later. The number of parameters of the Mamba depth sequence follows the budget described in Section 3.1.

**Mamba Placement Position.** Table 3 investigates the effect of inserting Mamba in different positions of the encoder-decoder architecture. The three configurations evaluated refer to the ResNet34 stage shown in Fig 1: Layer3 is the output of the third stage (256 channels at H/16 × W/16), Layer4 or bottleneck, is the output of the fourth stage (512 channels at H/32 × W/32), and Both indicates that it is inserted after Layer3 and Layer4 at the same time.

**Table 3:** Ablation on Mamba placement position under the simplified protocol (30 epochs, fixed learning rate). Positions refer to the ResNet34 stages marked in Fig 1. P2, that is, the bottleneck placement, gives the highest mIoU among the three evaluated Mamba placement strategies, so it is adopted by AquaMamba. These values are comparable only within this ablation table because the simplified protocol differs from the final training protocol used in Table 1.

Configuration	Position	Params	mIoU
P0: no Mamba (baseline)	—	29.16M	0.497
P1: after Layer3	Mid-level (256 × 32 × 32)	29.31M	0.510
P2: after Layer4	Deepest / bottleneck (512 × 16 × 16)	**29.52M**	**0.522**
P3: after Layer3 and Layer4	Both	30.40M	0.267 †

† Training was unstable in this configuration, the possible causes are discussed below.

Among the three evaluated strategies, the bottleneck placement (P2, 0.522 mIoU) has the highest accuracy rate, which is 0.012 mIoU higher than that of the middle placement (P1, 0.510mIoU). This is consistent with our design concept: at the bottleneck, the sequence of Mamba processing higher-level semantic features is four times shorter than that at Layer3, which may be conducive to improving computing efficiency while maintaining accuracy. Given that the results of the above two independent alation experiments are 0.005 mIoU, the difference between P1 and P2 is very small, so we regard the ranking of these two configurations as a reference rather than the final result.

In contrast, dual placement (P3) shows a substantial performance decrease (0.267 mIoU, −0.255 relative to P2), which was much greater than the 0.005 mIoU difference observed between two independent ablation runs. Under this configuration, the training is unstable. A reasonable explanation is that inserting two randomly initialized Mamba modules into the pretrained ResNet encoder at the same time may produce a large or conflicting gradient, thus interfering with the characterization of the pretrained. The NaN loss we observed when adding multiple new modules at the same time in the ASPP experiment (in Section 5.4) is consistent with this hypothesis. Since we have not inserted the training process, for example, by tracking the gradient norm of each module, the explanation has not been verified. The practical implication is deliberately limited to a smaller range: on a dataset of this size, the training of both single-point placement methods is stable, while the dual placement method is unstable. Among all the evaluated placement schemes, the mIoU value of the bottleneck placement scheme is the highest.

### 4.5. Unlabeled cross-domain confidence behavior

Since UIEB does not provide pixel-level semantic labels, we do not evaluate target-domain segmentation accuracy on UIEB [37]. Instead, we use UIEB only for an exploratory analysis of prediction confidence under an unlabeled domain shift. The goal is to describe whether the models preserve their average maximum softmax confidence when transferred from SUIM to UIEB, not to claim improved target-domain robustness.

Since UIEB does not provide pixel-level semantic annotations, direct IoU evaluation is not feasible. Instead, we use mean prediction confidence as a descriptive measure of model certainty. For each input image, the prediction confidence is computed as Eq (3):


$$\begin{array}{c} Confidence=\frac{\text{1}}{N}\sum_{i}max\_c\;p\left(c|x_{i}\right) \end{array}$$ (3)

where *N* denotes the total number of pixels, $x_{i}$ is the *i*-th pixel, and $p\left(c|x_{i}\right)$ is the softmax probability assigned to class *c* at pixel $x_{i}$*.* Here $x_{i}$ denotes an image pixel, distinct from the sequence token $x_{t}$ in Eq (1). This metric captures the average maximum class probability across all pixels: a model that produces sharp, decisive predictions yields high confidence, while a model that distributes probability mass across multiple categories yields low confidence. Confidence retention is then defined as the ratio of mean confidence on the target domain to that on the source domain, given by Eq (4):


$$\begin{array}{c} Retention\;=\;\frac{Confidence\_UIEB}{Confidence\_SUIM}\;\times \text{ 100}\% \end{array}$$ (4)

A higher retention value indicates that the model maintains a similar level of prediction confidence when transferred to the unlabeled target dataset. We emphasize that, because UIEB does not provide pixel-level semantic annotations, this metric measures the retention of prediction confidence under domain shift, not target-domain segmentation accuracy: a model could in principle be confidently wrong. We therefore treat the cross-domain results as preliminary evidence of stable prediction confidence on an unlabeled target dataset, rather than as direct evidence of prediction correctness. Results are presented in Table 4.

**Table 4:** Unlabeled cross-domain confidence behavior of representative architectures trained on SUIM and evaluated on UIEB: UNet (standard CNN), DeepLabV3+ (multi-scale CNN), Trans-UNet (Transformer bottleneck) and AquaMamba (SSM bottleneck). Values are mean maximum softmax confidence scores. Confidence retention is a descriptive confidence metric and should not be interpreted as target-domain segmentation accuracy. These values are comparable within this representative subset, not as a full ranking of all seven architectures in Table 1.

Method	SUIM	UIEB	Drop	Confidence retention
UNet	0.739	0.644	−0.095	87.1%
DeepLabV3+	0.838	0.737	−0.101	87.9%
Trans-UNet	0.913	0.821	−0.092	89.9%
**AquaMamba (Ours)**	**0.939**	**0.878**	**−0.061**	**93.5%**

**Notes**: Mean confidence is computed as the average maximum softmax probability across all pixels per image, then averaged over all images. Higher retention indicates preserved prediction confidence, not better target-domain segmentation accuracy.

**Key Result.** Within this four-model representative subset, AquaMamba retains the most prediction confidence under the unlabeled domain shift. AquaMamba retains 93.5% of its SUIM confidence on UIEB, while Trans-UNet, DeepLabV3+ and UNet retain 89.9%, 87.9% and 87.1% respectively. After incorporating Trans-UNet into the comparison, the result is consistent with the bottleneck-replacement comparison: under the same ResNet34-UNet setting, the Transformer-bottleneck model retains less confidence than AquaMamba but more than the two CNN baselines. This ordering reflects differences in prediction confidence retention on an unlabeled target dataset and should not be interpreted as evidence of higher target-domain segmentation accuracy. Fig 2(a) illustrates the changes in mean maximum softmax confidence from SUIM to UIEB across the four representative models, while Fig 2(b) compares their corresponding confidence retention rates.

**Dataset-specific interpretation.** In scenarios where the deployment conditions are different from SUIM, the results show that in addition to the intra-domain accuracy rate, confidence behavior should also be considered. However, due to the lack of semantic labeling in UIEB, the current analysis cannot determine whether a higher confidence retention rate corresponds to better target-domain segmentation accuracy.

### 4.6. Diagnostic analysis

To understand the nuanced performance differences within the selected representative subset, we conduct diagnostic analyses of AquaMamba, DeepLabV3+ and Trans-UNet based on object size and boundary-error rate. Objects are grouped as small (<1,024 px^2^), medium (1,024−10,000 px^2^) and large (>10,000 px^2^). Under these predefined thresholds, no ground-truth connected components fall within the medium-size interval. For category-level small-object reporting, categories with too few small connected components to support a stable class-level estimate are omitted; under this reporting rule, only RO is retained in the current analysis. We compare AquaMamba with the overall strongest baseline model DeepLabV3+ in Table 1 and with its controlled Transformer counterpart Trans-UNet so that the diagnostic analysis follows the CNN/Transformer/SSM paradigm framework introduced above. Results are summarized in Table 5 and Fig 3.

**Table 5:** Diagnostic analysis grouped by object size for AquaMamba, its Transformer counterpart Trans-UNet and DeepLabV3 + . Dashes indicate categories omitted from small-object reporting because the available small connected components in the SUIM test set were too sparse for a stable category-level estimate. Boundary-error rate is reported separately as mean ± per-image standard deviation: AquaMamba = 0.395 ± 0.122, Trans-UNet = 0.405 ± 0.112 and DeepLabV3+ = 0.448 ± 0.104; lower is better.

Class	Large Objects			Small Objects		
	AquaMamba	Trans-UNet	DeepLabV3+	AquaMamba	Trans-UNet	DeepLabV3+
HD	0.517	0.624	0.638	–	–	–
PF	0.324	0.345	0.393	–	–	–
WR	0.732	0.712	0.685	–	–	–
RO	0.771	0.741	0.707	0.096	0.445	0.479
RI	0.684	0.652	0.595	–	–	–
FV	0.713	0.723	0.618	–	–	–
SR	0.643	0.641	0.615	–	–	–

**Large Object Analysis**. Although DeepLabV3 + achieves the highest overall mIoU in Table 1, the large-object analysis in Table 5 shows complementary behavior among the three selected representative models. DeepLabV3 + obtains the highest IoU on HD (0.638, 0.624 for Trans-UNet and 0.517 for AquaMamba) and PF (0.393, 0.345 for Trans-UNet and 0.324 for AquaMamba). Since HD and PF are deformable categories, this pattern may be related to the multi-scale atrous convolutions in ASPP, although deformability itself was not measured quantitatively. Relative to DeepLabV3 + , AquaMamba is higher on five categories: FV (∆ = +0.095), RI (∆ = +0.089), RO (∆ = +0.064), WR (∆ = +0.047) and SR (∆ = +0.028). When Trans-UNet is included, AquaMamba leads in four categories (WR, RO, RI and SR), while Trans-UNet performs best on FV. Within the 7 large object categories reported for this subset, Trans-UNet has the highest mIoU, while AquaMamba leads in the largest number of individual categories. These categories have relatively coherent textures and context structures, and global-context modeling may contribute to the observed pattern by capturing broader scene-level information.

**Small object analysis**. In this diagnostic setting, only the RO category is retained for category-level small-object analysis. DeepLabV3 + achieves an IoU of 0.479, Trans-UNet achieves 0.445, and AquaMamba achieves 0.096. This result indicates that the current bottleneck-based, unidirectional AquaMamba implementation has clear limitations on the reported small-RO subset. Because Trans-UNet also uses the same 256-token bottleneck representation but maintains a higher IoU, this limitation cannot be attributed solely to down-sampling. Operation design, scanning direction and the bottleneck-only placement may also contribute.

**Boundary-error rate.** We compute the boundary error rate as the pixel-mismatch ratio within a 3-pixel dilated band around object boundaries. This region-based metric quantifies how many boundary-region pixels are misclassified and is distinct from the visual sharpness of an edge. AquaMamba achieves a lower boundary error than DeepLabV3+ (0.395 ± 0.122 vs. 0.448 ± 0.104, Δ = −0.053), with Trans-UNet in between (0.405 ± 0.112, Δ = −0.010 relative to AquaMamba). The similar boundary errors of AquaMamba and Trans-UNet suggest that the lower boundary error observed in this subset may be associated with bottleneck-level global-context modeling rather than uniquely with the SSM formulation. The residual advantage of AquaMamba over Trans-UNet may relate to selective SSM dynamics, calibration or other implementation details, which require separate investigation. We therefore interpret the lower boundary error as improved spatial consistency within boundary bands rather than as evidence of visually sharper contours. Complementary boundary-sensitive metrics such as Boundary IoU could provide additional insight in future work.

Overall, the diagnostic results within the selected representative subset indicate complementary architectural strengths rather than the uniform superiority of any single model. AquaMamba performs relatively well on large, contextually coherent categories and achieves a comparatively low boundary-region error. However, its current bottleneck-only, unidirectional configuration remains limited in the reported small object setting. These observations motivate the development of SSM-compatible multi-scale mechanisms and multi-resolution Mamba placement to improve the modeling of deformable objects and preserve fine-scale evidence.

### 4.7. Class-wise IoU difference analysis

Fig 4 presents class-wise IoU differences between AquaMamba and two representative baselines, Trans-UNet and DeepLabV3 + , based on the full SUIM test-set results reported in Table 1. The selected models represent the proposed SSM architecture, its controlled Transformer-bottleneck counterpart, and the strongest baseline in the full benchmark. This focused comparison complements the seven-model evaluation in Table 1 rather than replacing it.

Compared with the controlled Trans-UNet baseline, AquaMamba achieves higher IoU in five of the eight semantic categories: HD (+3.6 percentage points), PF (+8.1), WR (+1.4), RI (+1.4), and FV (+7.9). The largest positive differences occur in PF and FV. However, AquaMamba performs less well in BG (−1.1), RO (−3.1), and SR (−2.4), indicating that its advantage over the Transformer bottleneck is category-dependent rather than uniform. These findings are consistent with the overall mIoU difference of +1.9 percentage points between AquaMamba (0.669) and Trans-UNet (0.650), as reported in Table 1.

In contrast, DeepLabV3 + achieves higher IoU than AquaMamba across all eight categories, with the largest differences observed in PF (−6.9 percentage points) and SR (−5.6), expressed as AquaMamba minus DeepLabV3 + . This is consistent with the higher overall mIoU of DeepLabV3+ (0.712) compared with AquaMamba (0.669), indicating that the evaluated SSM bottleneck does not outperform the strongest multi-scale CNN baseline in this setting.

Overall, Fig 4 highlights class-dependent performance differences among the three representative architectures. Unlike the object-size and boundary-error diagnostics in Fig 3, the differences in Fig 4 are calculated from the class-wise IoU values for the full SUIM test set. These descriptive comparisons indicate that AquaMamba provides a favorable overall mIoU relative to the controlled Transformer-bottleneck baseline, while important limitations remain relative to DeepLabV3 + . The results should not be interpreted as evidence of uniform superiority across semantic categories or object scales.

## 5. Discussion

### 5.1. A signal-processing perspective on aquamamba’s behavior in underwater vision

The UIEB confidence retention pattern mode prompts us to interpret the signal processing of AquaMamba, although the experiments do not directly measure its internal filtering dynamics.

From a signal processing perspective, the state space equation ${\text{h}}_{\text{t}}=\overset{―}{\text{A}}\left({\text{x}}_{\text{t}}\right){\text{h}}_{\text{t}-\text{1}}+\overset{―}{\text{B}}\left({\text{x}}_{\text{t}}\right){\text{x}}_{\text{t}}$ implements an input-dependent infinite impulse response (IIR) filter. The discretized state-transition matrix $\overset{―}{\text{A}}\left({\text{x}}_{\text{t}}\right)$ of Eq (1) controls the “memory decay rate”, how quickly past information is forgotten, while $\overset{―}{\text{B}}\left({\text{x}}_{\text{t}}\right)$ gates the current input. Since both items depend on the input, the final operation can be interpreted as content adaptive, not spatially unchanged.

Underwater images suffer from three primary degradation mechanisms: wavelength-dependent absorption (red light attenuates first, creating blue-green color cast), forward scattering (light path deviation causing blur), and back-scattering (ambient light reflected by particles, reducing contrast). A standard convolution applies the same learning convolution kernel in all spatial positions, so it cannot effectively adapt to the degradation of spatial changes unless additional attention or multi-scale mechanisms are introduced. In contrast, Mamba can adjust its effective sequence processing behavior according to the spatial positions. Clearer regions may rely more on local information, while degraded or ambiguous regions may benefit from longer-range contextual information aggregation. This adaptive contextual modeling contributes to make more spatially coherent predictions near the boundary of the object.

This perspective is consistent with two observations from the selected analyses: AquaMamba remains competitive in several visually challenging categories and achieves the highest confidence retention in the unlabeled UIEB assessments. However, these observations do not prove that content adaptive filtering is the causal mechanism, because calibration, prediction sharpness and implementation details may also contribute. Therefore, Mamba provides a potentially effective balance at the compression bottleneck by combining global sequence modeling with linear sequence complexity. Table 6 summarizes the main differences among the three architectural paradigms.

**Table 6:** Simplified comparison of filtering mechanisms in the architectures. *L* denotes the sequence length (*L* = 256 at the bottleneck) and *K* the convolution kernel size.

Property	CNN	Transformer	Mamba
Filter type	Fixed	Content-adaptive	Content-adaptive
Receptive field	Local	Global	Global
Complexity	*O(L·K*^*2*^)	*O(L*^*2*^)	*O(L)*
Sequence representation	No explicit recurrent state	Pairwise attention map	Compressed recurrent state
Adaptation behavior	Spatially shared response	Content-adaptive pairwise interaction	Input-dependent state update

### 5.2. Complementary strengths: insights from diagnostic analysis

Although DeepLabV3 + achieves the highest overall mIoU in Table 1, the three-model diagnostic analysis in Section 4.6 reveals category- and scale-dependent strengths across DeepLabV3 + , Trans-UNet and AquaMamba. For large objects, AquaMamba ranks highest in 4 of the 7 categories (WR, RO, RI and SR), while Trans-UNet achieves the highest mIoU and performs best on FV. AquaMamba is also superior to DeepLabV3+ in 5 large object categories, among which the differences in FV, RI and RO are the most significant. These categories show relatively coherent textures and scene-level contextual structures, which may be beneficial to global-context modeling.

The boundary-error rate of AquaMamba is also 0.053 lower than that of DeepLabV3 + , indicating better spatial consistency in the evaluated boundary band. However, because Trans-UNet performs similarly to AquaMamba on this metric, the observed reduction is more likely associated with bottleneck-level global-context modeling than with content-adaptive SSM gating alone. Therefore, the current evidence does not prove that Mamba’s unique mechanism is the only reason for the lower boundary-error rate.

In contrast, DeepLabV3 + performs best on HD and PF, which may benefit from the multi-scale feature extraction provided by the ASPP module. Because deformability was not measured quantitatively, this explanation remains preliminary. AquaMamba’s performance on the reported small-RO subset is also substantially lower, indicating that its current bottleneck-only and unidirectional configuration may not fully preserve isolated fine-scale evidence. However, because only the RO category was retained for category-level small-object reporting under the predefined reporting rule, this result should not be generalized to all small-object categories or to all SSM-based segmentation models.

Overall, the diagnostic results show complementary advantages among the selected architectures rather than absolute superiority of a single model. AquaMamba is comparatively effective in several large and contextually coherent categories and achieves a low boundary-region error, while DeepLabV3 + maintains advantages in deformable categories. These findings motivate hybrid architectures that combine SSM-based global-context modeling with CNN-based multi-scale processing and high-resolution feature retention.

### 5.3. Why multi-scale remains critical

The diagnostic results suggest two possible contributors to the overall mIoU gap between AquaMamba and DeepLabV3 + : weaker performance on HD/PF and poor performance on the reported small-RO subset. However, the present analysis does not quantify the exact contribution of each subset to the overall mIoU difference. The comparison with Trans-UNet further suggests that down-sampling alone is insufficient to explain the small-RO weakness, because the Transformer bottleneck uses the same 256-token representation.

### 5.4. Multi-scale integration attempts and lessons learned

We attempted to integrate ASPP directly into AquaMamba through two strategies, both yielding negative results under the tested configurations.

In the first approach, an end-to-end Mamba-ASPP-UNet with 42.34M parameters placed Mamba modules at both Layer3 and Layer4 and inserted ASPP after Layer4. Under the standard settings ($lr=\text{3}\times {\text{10}}^{-\text{4}}$, AdamW) the loss became NaN from the first epoch, and the model collapsed to predicting only the background class. One possible explanation is that simultaneously introducing multiple randomly initialized modules caused optimization instability and disrupted the pretrained ResNet representations, although this mechanism was not directly examined.

In the second approach, pretrained AquaMamba weights were used for incremental ASPP integration. During Phase 1, the existing parameters of AquaMamba were frozen, and only the newly added ASPP module and gating fusion layer were trained, containing a total of 9.44M trainable parameters. This phase achieved an mIoU of 0.550. During Phase 2, the full network was unfrozen and fine-tuned using differential learning rates, resulting in an mIoU of 0.668, which remained marginally below the original AquaMamba result of 0.669.

These experiments indicate that it is not enough to directly combining ASPP with bottleneck-level SSM features under the evaluated configurations. Possible contributing factors include: a mismatch between globally contextualized Mamba features and the locally aggregated multi-scale features produced by ASPP, an increased risk of overfitting due to the larger model capacity relative to the 1,525-image training set, and potential redundancy between Mamba state propagation and the global pooling branch of ASPP. Therefore, the future multi-scale SSM architecture may require a more tightly integrated design, such as applying SSM scanning at specific scale state dimension or scan step length at multiple resolution levels instead of using ASPP as an additional parallel branch.

### 5.5. Dataset-specific observations and design considerations

SUIM provides in-domain segmentation results with labels, while UIEB only supports confidence-based cross-domain analysis because it lacks pixel-level annotations. Therefore, AquaMamba’s higher confidence retention on UIEB should not be interpreted as evidence of higher target-domain segmentation accuracy. In addition, the small-object result is based on the reported small-RO subset and cannot be generalized to all small-object categories. The comparison with Trans-UNet suggests that down-sampling alone cannot explain this limitation, because both models use the same 256-token bottleneck representation.

### 5.6. Limitations

Several limitations of this study should be acknowledged. First, all models are trained and evaluated in-domain on a single dataset, SUIM, and results may vary on larger or domain-specific underwater datasets. The best-performing Mamba configuration likely depends on dataset scale and characteristics.

Second, our cross-domain evaluation uses mean prediction confidence in Eq (3) as a descriptive measure because UIEB lacks pixel-level semantic annotations. While this metric captures prediction decisiveness, it does not directly measure segmentation accuracy; a model could produce confident but incorrect predictions. Future work should develop annotated cross-domain underwater segmentation benchmarks to enable direct IoU-based evaluation of generalization performance.

Third, the extended analyses do not cover all evaluated architectures uniformly. The object-size and boundary-error diagnostics (Table 5 and Fig. 3), together with the class-wise IoU difference analysis (Fig. 4), include AquaMamba, DeepLabV3 + , and Trans-UNet, whereas the cross-domain confidence analysis (Table 4 and Fig 2) additionally includes UNet. UNet++, FPN and MA-Net are not included in these extended analyses. The object size coverage is also limited: only the RO category is retained for small object category-level reporting, and no ground-truth components fall within the predefined medium-size range of 1,024−10,000 px^2^. Consequently, the current results cannot provide a complete architecture-wide or scale-wide comparison.

Fourth, our current Mamba implementation processes flattened 2D features as a 1D sequence, which may limit the preservation of two-dimensional spatial relationships. Bidirectional or 2D-aware scanning approaches, such as those employed in Vision Mamba [10], may improve performance by better preserving spatial relationships.

Finally, this study focuses on single-image segmentation without exploiting temporal information. Future work may extend SSM-based methods to underwater video to improve temporal consistency.

## 6. Conclusion

This study investigates selective SSMs for underwater semantic segmentation under a controlled ResNet34-UNet framework. Across seven methods on the SUIM benchmark, AquaMamba outperforms the constructed Transformer bottleneck by 1.9 percentage points in mIoU while using fewer parameters, indicating a favorable parameter-accuracy trade-off under the evaluated setting. DeepLabV3 + achieves the highest overall mIoU of 0.712, highlighting the continuing importance of multi-scale feature extraction.

Within the selected representative subset, AquaMamba also achieves the highest confidence retention on the unlabeled UIEB dataset. However, this result reflects predictive confidence under domain shift rather than target-domain segmentation accuracy, and it should be verified on annotated cross-domain datasets.

The diagnostic analyses further reveal complementary architectural advantages rather than uniform superiority. AquaMamba performs well on several large, contextually coherent categories and achieves low boundary-region error, but its current bottleneck-only, unidirectional configuration remains limited on the reported small-RO subset. These findings motivate future architectures that combine SSM-based global-context modeling with multi-scale and high-resolution feature preservation.
